# Pharmacokinetics of high-dose tigecycline in critically ill patients with severe infections

**DOI:** 10.1186/s13613-020-00715-2

**Published:** 2020-07-13

**Authors:** Gennaro De Pascale, Lucia Lisi, Gabriella Maria Pia Ciotti, Maria Sole Vallecoccia, Salvatore Lucio Cutuli, Laura Cascarano, Camilla Gelormini, Giuseppe Bello, Luca Montini, Simone Carelli, Valentina Di Gravio, Mario Tumbarello, Maurizio Sanguinetti, Pierluigi Navarra, Massimo Antonelli

**Affiliations:** 1 Dipartimento di Scienza dell’Emergenza, Anestesiologiche e della Rianimazione - UOC di Anestesia, Rianimazione, Terapia Intensiva e Tossicologia Clinica, Fondazione Policlinico Universitario A. Gemelli IRCCS-Università Cattolica del Sacro Cuore, Largo A. Gemelli 8, 00168 Rome, Italy; 2 Istituto di Anestesia e Rianimazione, Università Cattolica del Sacro Cuore, Rome, Italy; 3 Institute of Farmacologia, Università Cattolica del Sacro Cuore, L.go F. Vito 1, Rome, Italy; 4 Fondazione Policlinico Universitario Agostino Gemelli IRCCS, Rome, Italy; 5 Dipartimento di Scienze di Laboratorio e Infettivologiche, UOC di Malattie Infettive, Fondazione Policlinico Universitario A. Gemelli IRCCS, Rome, Italy; 6 Istituto di Malattie Infettive, Università Cattolica del Sacro Cuore, Rome, Italy; 7 Istituto di Microbiologia, Università Cattolica del Sacro Cuore, Rome, Italy; 8 Dipartimento di Scienze di Laboratorio e Infettivologiche, UOC di Microbiologia, Fondazione Policlinico Universitario A. Gemelli IRCCS, Rome, Italy

**Keywords:** Tigecycline, High dose, Pharmacokinetics, Epithelial lining fluid, Critically ill patients, Severe infections

## Abstract

**Background:**

In critically ill patients, the use of high tigecycline dosages (HD TGC) (200 mg/day) has been recently increasing but few pharmacokinetic/pharmacodynamic (PK/PD) data are available. We designed a prospective observational study to describe the pharmacokinetic/pharmacodynamic (PK/PD) profile of HD TGC in a cohort of critically ill patients with severe infections.

**Results:**

This was a single centre, prospective, observational study that was conducted in the 20-bed mixed ICU of a 1500-bed teaching hospital in Rome, Italy. In all patients admitted to the ICU between 2015 and 2018, who received TGC (200 mg loading dose, then 100 mg q12) for the treatment of documented infections, serial blood samples were collected to measure steady-state TGC concentrations. Moreover, epithelial lining fluid (ELF) concentrations were determined in patients with nosocomial pneumonia. Amongst the 32 non-obese patients included, 11 had a treatment failure, whilst the other 21 subjects successfully eradicated the infection. There were no between-group differences in terms of demographic aspects and main comorbidities. In nosocomial pneumonia, for a target AUC_0-24_/MIC of 4.5, 75% of the patients would be successfully treated in presence of 0.5 mcg/mL MIC value and all the patients obtained the PK target with MIC ≤ 0.12 mcg/mL. In intra-abdominal infections (IAI), for a target AUC_0-24_/MIC of 6.96, at least 50% of the patients would be adequately treated against bacteria with MIC ≤ 0.5 mcg/mL. Finally, in skin and soft-tissue infections (SSTI), for a target AUC_0-24_/MIC of 17.9 only 25% of the patients obtained the PK target at MIC values of 0.5 mcg/mL and less than 10% were adequately treated against germs with MIC value ≥ 1 mcg/mL. HD TGC showed a relevant pulmonary penetration with a median and IQR ELF/plasma ratio (%) of 152.9 [73.5–386.8].

**Conclusions:**

The use of HD TGC is associated with satisfactory plasmatic and pulmonary concentrations for the treatment of severe infections due to fully susceptible bacteria (MIC < 0.5 mcg/mL). Even higher dosages and combination strategies may be suggested in presence of difficult to treat pathogens, especially in case of SSTI and IAI.

## Background

Tigecycline (TGC), the first antimicrobial of glycilcycline class, has shown an expanded-spectrum activity against gram-positive, gram-negative, aerobic, anaerobic and atypical bacterial species, including antibiotic-resistant strains [1]. Indeed, it has been demonstrated that methicillin-resistant *Staphylococcus aureus* (MRSA), penicillin-resistant *Streptococcus pneumoniae*, vancomycin-resistant *enterococci* (VRE), extended-spectrum β-lactamase (ESBL)/carbapenem-producing *Enterobacterales* and extensively drug-resistant (XDR) *Acinetobacter baumannii* are susceptible to TGC [2–4].

TGC is currently approved by the U.S. Food and Drug Administration (FDA) for complicated skin and skin-structure infections, complicated intra-abdominal infections, community-acquired pneumonia with an initial dose of 100 mg, followed by 50 mg every 12 h. Nevertheless, due to an increased risk of death compared to other antimicrobials, its use has recently been restricted in situations when alternative treatments are not suitable ([5], https://www.fda.gov/drugs/drug-safety-and-availability/fda-drug-safetycommunication-fda-warns-increased-risk-death-iv-antibacterial-tygacil-tigecycline).

However, the alarming increase in antimicrobial resistance amongst the nosocomial pathogens is leading the clinicians to consider the use of TGC as an important therapy in the management of difficult to treat infection, particularly in critically ill patients. This is also supported by recent studies suggesting that previous failures of TGC therapy in critically ill patients were likely due to a drug underdosage [6, 7] and that standard doses provide serum concentrations that are below the minimum-inhibitory concentrations (MICs) of most MDR pathogens. Moreover, it has been reported an increased effectiveness of high-dose TGC (HD TGC) regimen to improve the clinical outcome, without safety issues [8–11].

Therefore, we designed this prospective observational study to describe the pharmacokinetic/pharmacodynamic (PK/PD) profile of HD TGC in a cohort of critically ill patients with severe infections.

## Methods

### Patients and study design

This was a prospective, observational study that was performed between 2015 and 2018 in the 20-bed ICU of a 1500-bed teaching hospital in Rome, Italy. The protocol was approved by the Catholic University’s Ethical Committee (approval number Prot.sf 8431/13). Written informed consent was obtained from the patients’ legally authorized representative. Critically ill adult patients were considered eligible for the study when the attending physician prescribed TGC as empirical treatment (within 12 h from microbiological sampling) of a possible MDR infection, or as targeted therapy based on definitive results, in the absence of any exclusion criteria: known TGC allergy, creatinine clearance less than 40 mL/min (calculated according to the Cocrockft–Gault formula) apart from those ones who were anuric and on continuous renal replacement therapy (CRRT), hyperbilirubinemia (bilirubin level higher than 3 mg/dL), severe hepatic failure (Child–Pugh C), little chance of survival as defined by the Simplified Acute Physiology 2 (SAPS 2) score > 80, concomitant treatment with other drugs that can potentially interfere with TGC (i.e., rifampin and cyclosporine). Patient without microbiologically confirmed infection were not excluded. TGC was administered intravenously at loading dose (LD) of 200 mg over 30-min, followed by 100 mg over 30-min bid. On day 4 after the commencement of the HD TGC, at steady state, pharmacokinetic analyses of the study group were performed. Clinical and demographic data were recorded upon enrolment. Safety and adverse events were determined through the observed biochemical abnormalities, documented according to the Department of Health and Human Services–Common Terminology Criteria for Adverse Events (DHHS-CTCAE v.3.0) classification [12].

Clinical cure was defined as the complete resolution of all signs and symptoms of the infection by the end of TGC therapy. In case of ventilator-associated pneumonia (VAP), improvement or lack of progression of all abnormalities on chest radiographs was also required [13]. Otherwise, the outcome was classified as treatment failure. Clinical outcomes were independently evaluated by two physicians (GDP, MSV) when judgments were discordant (two cases), the reviewers reassessed the data and reached a consensus decision. The quality of source control was considered adequate when it included drainage of infected fluid collections, debridement of infected solid tissue, removal of devices/foreign bodies, and definitive measures to correct anatomic derangements resulting in on-going microbial contamination and to restore optimal function within 48 h after diagnosis [14].

### Sample collection

Although TGC concentration–time profiles are stable dose just on day 3, blood samples were collected after the seventh dose (on day 4 of treatment) at T0 (immediately before the initiation of the infusion) and 1, 1.5, 2, 4, 6, 8, 10, and 12 after the start of infusion. According to patients’ respiratory status, one mini-bronchoalveolar lavage (BAL) (40 mL sterile 0.9% saline solution was blindly instilled through a telescopic catheter and immediately aspirated in a trap) was performed on day 4, in case of suspected HAP.

### Preparation of stock solution and calibration standard

Stock solution of TGC and the internal standard (IS), propranolol hydrochloride, were prepared by dissolving accurately weighed amounts of each compound in MeOH to obtain a final concentration of 0.1 mcg/mL. Calibration standards were prepared by diluting stock solutions of TGC in drug-free human plasma to yield TGC concentrations of 5000, 2500, 1250, 625, 312.5, 156.25, 78.125, 39.1, 19.5 and 9.76 ng/mL.

### Sample preparation

Tigecycline liquid/liquid extraction from plasma samples (see Additional file 1).

Tigecycline solid-phase extraction from BAL samples (see Additional file 1).

Chromatographic and Mass-Spectrometric Conditions (see Additional file 1)

### Urea assay

#### Determination of urea in plasma and BAL samples

Urea levels were detected by the QuantiChrom Urea Assay kit (BIOassay System, Hayward, CA, USA), which was used according to the manufacturer’s instructions.

### Pharmacokinetic/pharmacodynamics analysis

A one-compartment model with first-order elimination determined pharmacokinetic parameters. The 0–12 h area under the time–concentration curve (AUC_0–12_) was determined by the linear trapezoidal rule. TGC AUC_0–24_ was calculated as AUC_0–12_ X 2. TGC maximum and minimum concentrations (*C*_max_, *C*_min_) were directly obtained from observed peak and trough concentrations. Epithelial lining fluid (ELF) tigecycline (TGC_ELF_) concentration was calculated from BAL concentration (TGC_BAL_) using urea as dilution marker: TGC_ELF_ = TGC_BAL_ X urea dilution index (plasma urea concentration/BAL urea concentration) [15]. In all patients, distribution volume (Vd), drug clearance (CL), and elimination half-life (*t*_1/2_) were calculated after a single 100-mg intravenous dose at steady state.

According to previous literature, based on early animal efficacy studies using a classification and regression tree approach, area under the concentration curve (AUC) _0–24_/MIC ratio ≥ 4.5, 6.96 and 17.9 were used as PD targets for VAP, intra-abdominal infections (IAI) and skin–soft-tissue infections (SSTI), respectively [6]. Graphing of data was undertaken using Prism version 6.0 for Windows (graphPad Software, San Diego, CA).

### Microbiological analysis

Isolates were identified by matrix-assisted laser desorption ionization-time-of-flight (MALDI-TOF) mass spectrometry (MALDI Biotyper, Bruker Daltonics GmbH, Leipzig, Germany). The in vitro susceptibility of the isolates was assessed with the Vitek 2 system (bioMérieux, Marcy l’Etoile, France) or with panels manufactured by MERLIN Diagnostica GmbH (Bornheim, Germany). Results were interpreted in accordance with the European Committee on Antimicrobial Susceptibility Testing (EUCAST) clinical breakpoints. The presence of carbapenemase genes of blaKPC, blaNDM, blaVIM, blaOXA-48, blaOXA-23, and blaOXA-58 types was determined by polymerase chain reaction and DNA sequencing analysis using previously described protocols (Endemiani, Poirel, Woodford) [16].

### Statistical analysis

All statistical analyses were performed using MedCalc software, version 12.2.1 (MedCalc^®^, MariaKerke, Belgium). Kolmogorov–Smirnov test was used to value the variables distribution. The data with a non-Normal distribution were assessed with Mann–Whitney test and the median and selected centiles’ (25th–75th) value were given (interquartile range, IQR). The data with a Normal distribution were assessed with Student’s test. Categorical variables are presented as proportions and were analysed with the use of the Chi square test or Fisher’s exact test, as appropriate. A *p* value < 0.05 was considered significant. Due to the PK/PD design of the study, a sample size was not calculated, foreseeing the recruitment of at least 30 patients during the predefined study period (July 2015–July 2017).

## Results

### Patients’ characteristics

The clinical details of the 32 non-obese patients included in the study are listed in Table 1. Albumin levels were quite low with an overall positive fluid balance at enrolment. Median SAPS II score was 53.5 and the most relevant comorbidities were cardiovascular diseases, chronic obstructive pulmonary disease, chronic renal failure and neoplasm (Table 1). Median SOFA score was 7 and many patients were in septic shock or presented with acute respiratory failure (ARF) and acute kidney injury (AKI) requiring mechanical ventilation (MV) and CRRT, respectively. More than half of the patients had VAP, followed by intra-abdominal infections and skin and soft-tissue infections: in the microbiological case-mix Gram-negative bacteria were mostly represented (90.6%). Median duration of TGC therapy was 12 days and it was started empirically in half of the cases. The use of vasopressors and MV during TGC therapy was high and 30-day mortality rate was 28.1%. Eleven patients had a treatment failure, whilst the other 21 successfully eradicated the infection. There were no between-group differences in terms of demographic aspects and main comorbidities. Further the two groups were similar in terms of presenting features and outcomes with the exception of VAP rate which was higher in the treatment success group (76.2% vs. 27.3%, *p *= 0.02) and a trend to a higher percentage of skin and soft-tissue infections and source control amongst patients who failed TGC treatment (*p *= 0.06 and *p *= 0.07, respectively).

**Table 1:** Baseline patients’ characteristics

	Total cohort (*n* = 32)	Treatment failure (*n* = 11)	Treatment success (*n* = 21)	*p* value
Demographics and comorbidities				
Age, years	56 [46–68.5]	55 [49.75–71]	56 [45–68.25]	0.75
Male sex, N (%)	17 (53.1)	5 (45.5)	12 (57.1)	0.8
Weight, (kg)	76.5 [60–90]	75 [67.8–80]	90 [60–100]	0.45
Albumin, (g/dL)*	23 [21.5–26.5]	22 [19.25–26.25]	24 [22.75–26.5]	0.17
Fluid balance, (mL)*	+762.9 [−393 to +3703.5]	+3332 [−1124.2 to + 4112]	616.3 [−358.5 to + 2592.7]	0.5
SAPS II score	53.5 [44.5–67.5]	61 [44.7–66.5]	52 [43.5–67.5]	0.92
Cardiovascular diseases, N (%)	6 (18.75)	3 (27.3)	3 (14.3)	0.39
COPD, N (%)	5 (15.6)	1 (9.1)	4 (19.1)	0.64
Chronic renal failure, N (%)	7 (21.9)	3 (27.3)	4 (19.1)	0.4
Diabetes, N (%)	3 (9.4)	0	3 (14.3)	0.53
Neoplasm, N (%)	7 (21.9)	4 (36.4)	3 (14.3)	0.2
Presenting features and outcomes				
ICU LOS before TGC, (days)	7.5 [2.5–16]	5 [0.5–11.25]	12 [3.75–18.25]	0.13
MV duration before TGC (days)	8 [3–12]	5 [0.5–11.25]	8 [3.75–14.75]	0.19
Vasopressors duration before TGC (days)	4.5 [0–8.5]	5 [0.25–8.25]	4 [0–8.25]	0.89
SOFA score*	7 [4–10]	8 [4.75–12]	6 [4–9]	0.2
Septic shock, N (%)*	18 (56.3)	7 (63.6)	11 (52.4)	0.71
ARF requiring MV, N (%)*	28 (87.5)	10 (90.9)	18 (85.7)	1
AKI requiring CRRT, N (%)*	11 (34.4)	3 (27.3)	8 (38.1)	0.7
Creatinine clearance (ml/min)*	97.3 [32–150.8]	63.2 [32–155]	104 [30–142]	0.85
VAP, N (%)	19 (59.4)	3 (27.3)	16 (76.2)	*0.02*
Non-pulmonary infections, N (%)#	13 (40.6)	8 (72.7)	5 (23.8)	*0.02*
Secondary bacteraemia, N (%)	13 (40.6)	4 (36.4)	9 (42.9)	1
Source control, N (%)	13 (40.6)	7 (63.6)	6 (28.6)	*0.07*
TGC therapy duration,(days)	12 [9–15]	12 [10–15]	11 [8–17]	0.69
TGC empirical therapy, N (%)	17 (53.1)	7 (63.6)	10 (47.6)	0.47
Gram-positive bacteria N (%)**	11 (34.4)	4 (36.4)	7 (33.3)	1
Gram-negative bacteria N (%)***	29 (90.6)	10 (90.9)	19 (90.5)	1
ICU LOS after TGC, (days)	15 [10.5–27]	14.5 [12–19]	16 [10–31.4]	0.42
MV duration after TGC (days)	10 [5–15]	14 [9.75–15.75]	8 [2–13.5]	0.04
Vasopressors duration after TGC (days)	3 [1.5–13]	8 [2.25–13]	3 [0–10.75]	0.12
30-day mortality	9 (28.1)	8 (72.7)	1 (4.8)	< 0.001

Data are presented as median [IQR], unless otherwise indicated

*Pts* patients, *VAP* ventilator-associated pneumonia; *TGC* tigecycline, *SAPS II* Simplified Acute Physiology Score, *COPD* chronic obstructive pulmonary disease, *LOS* length of stay, *ICU* Intensive Care Unit, *MV* mechanical ventilation, *SOFA* Sequential Organ Failure Assessment, *AKI* acute kidney injury; *CRRT* continuous renal replacement therapy, *ARF* acute respiratory failure, *MV* mechanical ventilation; *kg* kilogram, *IQR* interquartile range

* Evaluated at TGC starting day

** i.e. *Staphylococcus aureus (n *= 6), *enterococci (n *= 3)*, streptococcus* spp. *(n *= *2)*

*** i.e. *Acinetobacter baumannii (n *= *10)*, carbapenem-resistant *Klebsiella pneumonia (n *= *6)*, *Escherichia coli (n *= *6)*, *Proteus* spp*. (n *= *5), Bacteroides* spp*. (n *= *2)*

^#^ Ten intra-abdominal infections and three skin and soft-tissue infections

### Pharmacokinetic results

**Fig. 1:**
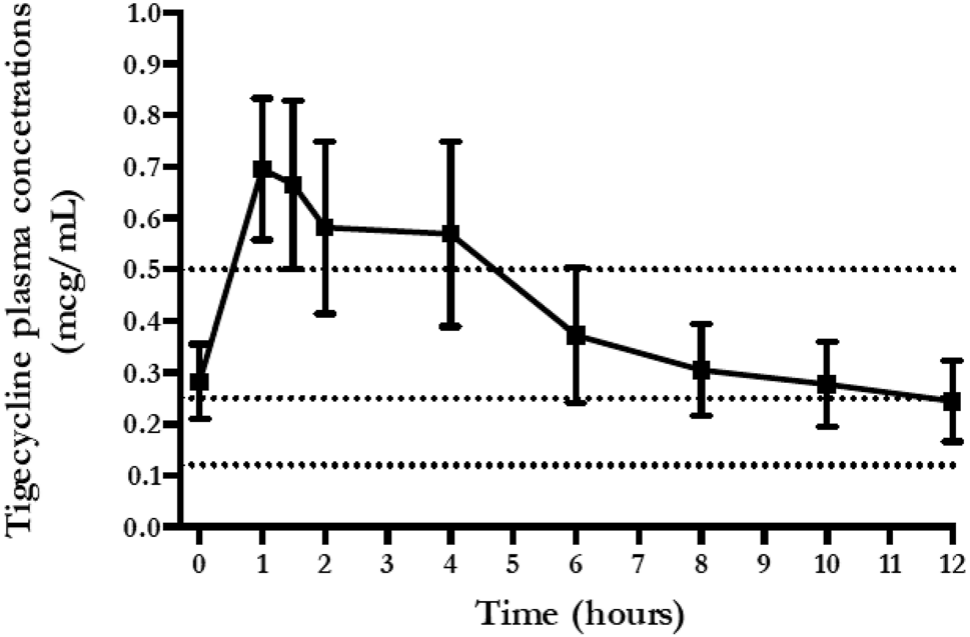
Total tigecycline plasma concentration (mean ± SE) versus time of administration

**Fig. 2:**
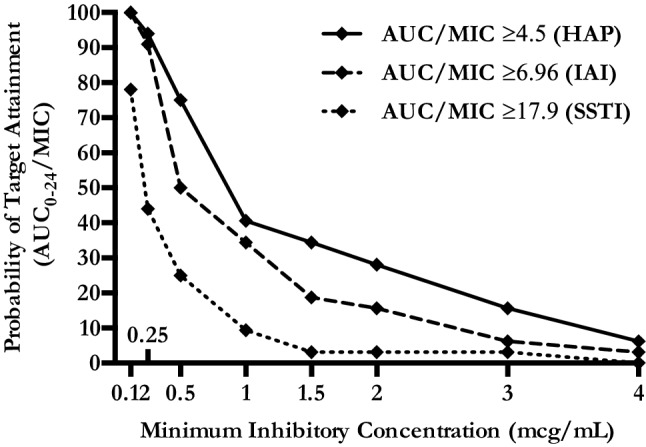
Probability of target attainment of pharmacodynamics indices in plasma, according to infection types and MIC. HAP: hospital-acquired pneumonia; IAI: intra-abdominal infection; SSTI: skin and soft-tissue infection; AUC: area under the curve; MIC: minimum inhibitory concentration (mcg/mL)

A one-compartment model with first-order disposition processes adequately described the concentration–time curve, although significant interindividual variability was observed. *Vd*, Cl and t_1/2_ were 438.6 L, 42.1 L/h and 7.2 h, respectively. Median and IQR values of *C*_max_ and *C*_min_ were 0.34 [0.15–1.03] mcg/mL and 0.09 [0.05–0.26] mcg/mL (Table 1). Figure 1 shows the mean ± SD time–concentration profile at different time points of plasma tigecycline concentrations, compared with most frequently observed MIC values (0.12–0.25–0.5 mcg/mL). AUC_0-24_ and IQR were calculated for each patient and the percentage of target attainment was also computed for nosocomial pneumonia (NP) (AUC_0-24_/MIC breakpoint of 4.5), complicated intra-abdominal infections (cIAI) (AUC_0-24_/MIC breakpoint of 6.96) and complicated skin and soft-tissue infections (cSSTI) (AUC_0-24_/MIC breakpoint of 17.9). Considering a target AUC_0-24_/MIC of 4.5, 75% of the patients would be successfully treated in presence of 0.5 mcg/mL MIC value and all the patients obtained the PK target with MIC ≤ 0.12 mcg/mL. Considering a target AUC_0-24_/MIC of 6.96, at least 50% of the patients would be adequately treated against bugs with MIC ≤ 0.5 mcg/mL, whilst only 15.6% obtained the PK target with MIC of 2 mcg/mL. Finally, with a target AUC_0-24_/MIC of 17.9 only 25% of the patients obtained the PK target at MIC values of 0.5 mcg/mL and less than 10% were adequately treated against germs with MIC value ≥ 1 mcg/mL (Fig. 2).

### Pulmonary concentrations

**Fig. 3:**
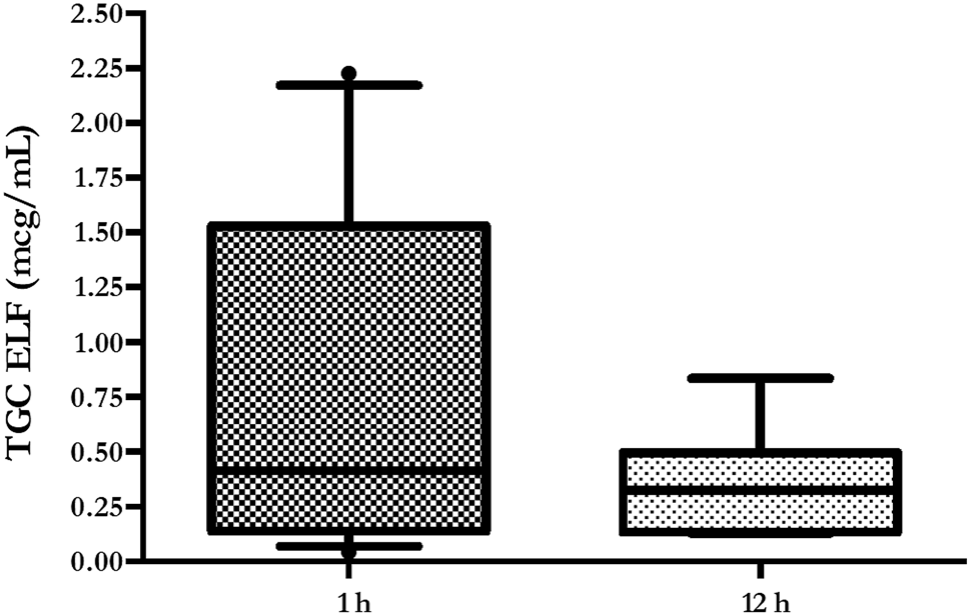
Boxplot showing tigecycline ELF concentrations. Boxes represent interquartile ranges (lower border 25th percentile; upper border 75th percentile), and the horizontal lines within the boxes indicate the medians (50th percentile). Whiskers indicate minimum and maximum values

**Fig. 4:**
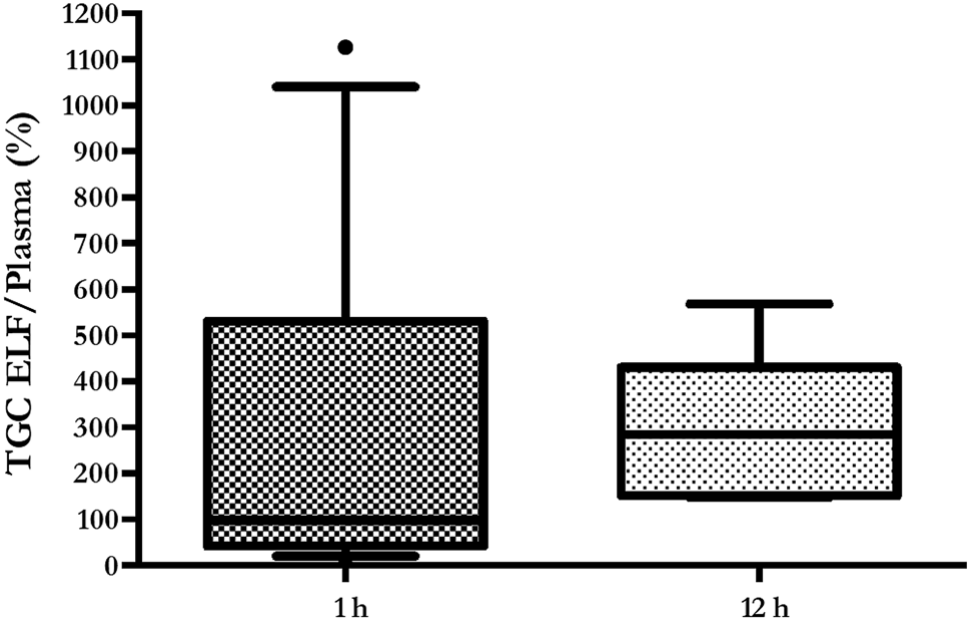
Boxplot showing tigecycline ELF to plasma ratio (%). Boxes represent interquartile ranges (lower border 25th percentile; upper border 75th percentile), and the horizontal lines within the boxes indicate the medians (50th percentile). Whiskers indicate minimum and maximum values

Tigecycline pulmonary concentrations were measured in 12 (1 h) and 7 (12 h) patients, respectively. Main reasons to exclude samples were presence of blood and excessive dilution, whilst five patients did not undergo BAL due to severe respiratory failure. Median and IQR ELF *C*_max_ was 0.42 [0.15–1.2] and ELF *C*_min_ was 0.32 [0.17–0.43]; median and IQR ELF/plasma ratio (%) was 152.9 [73.5–386.8] (Table 2). Mean ± SE ELF concentrations were similar at 1 h and 12 h (0.78 ± 0.2 mcg/mL vs. 0.36 ± 0.1 mcg/mL; *p* = 0.19) (Fig. 3). Conversely, no significant differences were found comparing mean ± SE ELF/plasma ratio at 1 h and 12 h (281 ± 107.6 vs. 298.3 ± 60.7; *p* = 0.9) (Fig. 4).

**Table 2:** Steady-state serum and alveolar TGC PK parameters in the 32 enrolled patients

Parameter	Patients (*n* = 32)
*Vd*, L	438.6
CL, L/h	42.1
t_1/2_, h	7.2
*C*_max_, mcg/mL	0.34 [0.15–1.03]
*C*_min_, mcg/mL	0.09 [0.05–0.26]
ELF *C*_max_, mcg/mL*	0.42 [0.15–1.2]
ELF *C*_min_, mcg/mL*	0.32 [0.17–0.43]
ELF/plasma ratio (%), median [IQR]*	152.9 [73.5–386.8]
AUC_0-24_, mcg h/mL	3.61 [2.55–10.39]
AUC_0-24_/0.12 mcg/mL MIC ≥ 4.5, (%)	100
AUC_0-24_/0.25 mcg/mL MIC ≥ 4.5, (%)	94
AUC_0-24_/0.5 mcg/mL MIC ≥ 4.5, (%)	75
AUC_0-24_/1 mcg/mL MIC ≥ 4.5, (%)	40.6
AUC_0-24_/2 mcg/mL MIC ≥ 4.5, (%)	28.1
AUC_0-24_/0.12 mcg/mL MIC ≥ 6.96, (%)	100
AUC_0-24_/0.25 mcg/mL MIC ≥ 6.96, (%)	91
AUC_0-24_/0.5 mcg/mL MIC ≥ 6.96, (%)	50
AUC_0-24_/1 mcg/mL MIC ≥ 6.96, (%)	34.4
AUC_0-24_/2 mcg/mL MIC ≥ 6.96, (%)	15.6
AUC_0-24_/0.12 mcg/mL MIC ≥ 17.9, (%)	78
AUC_0-24_/0.25 mcg/mL MIC ≥ 17.9, (%)	44
AUC_0-24_/0.5 mcg/mL MIC ≥ 17.9, (%)	25
AUC_0-24_/1 mcg/mL MIC ≥ 17.9, (%)	9.4
AUC_0-24_/2 mcg/mL MIC ≥ 17.9, (%)	3.1

Data are expressed as median [IQR] and N (%)

*TGC* tigecycline; *PK* pharmacokinetic; *Vd* volume of drug distribution, *IQR* interquartile range; *CL* drug clearance; *t*_*1/2*_ elimination half-life; *C*_max_ peak plasmatic concentration; *C*_min_ trough plasmatic concentration; *ELF* epithelial lining fluid; *MIC* minimum inhibitory concentration; *AUC* total drug area under the time–concentration curve

*TGC ELF concentrations were measured in 12 (1 h) and 7 (12 h) samples, respectively

## Discussion

Our study shows an HD TGC (200 mg LD, then 100 mg q12) time–curve concentration with mean peak and trough levels of 0.65 mcg/mL and 0.25 mcg/mL, respectively (Fig. 1). AUC_0-24_/MIC targets for nosocomial pneumonia (≥ 4.5) and complicated intra-abdominal infections (≥ 6.96) were obtained in the majority of cases in presence of bacteria with MIC values ≤ 0.25. Otherwise, lower MIC values (≤ 0.12 mcg/mL) were required to have satisfactory AUC_0-24_/MIC results (78%), whilst treating a skin/soft-tissue infection (Fig. 2, Table 2). Similar to plasma 1 h and 12 h, pulmonary concentrations (0.78 mcg/mL and 0.36 mcg/mL, respectively) were observed with a good median ELF/plasma ratio of 152.9% (Table 2, Fig. 3). This high-dose regimen was associated with a 65.6% of treatment success rate in a normal weight population including 60% of VAP, 31% of cIAI and 9% of SSTI. TGC was used in half of the cases as targeted regimen for a median duration of 12 days. The rates of septic shock, acute respiratory failure requiring MV and acute kidney injury requiring CRRT were also high, with a mortality rate of 28.1% (Table 1).

The pharmacokinetics/pharmacodynamics and tissue penetration of tigecycline have been extensively studied in various in vitro and human models [17]. However, these studies were generally carried out in healthy volunteers, and few pharmacokinetic data concerning infected patients are available, which may present pathophysiologic conditions influencing the pharmacokinetic profile of this molecule. In addition, the majority of available data in infected patients derive from studies where normal doses are used, although for severe nosocomial infections a double-dose regimen is warranted [18, 19].

Recently, standard-dose TGC pharmacokinetics in ten critically ill patients has been studied [6]. The authors observed that a larger body mass index was associated with increased TGC *Cl*, but standard doses produced satisfactory plasmatic levels for VAP and cIAI treatment due to *Enterobacter cloacae*, *Esherichia coli*, *Klebsiella pneumoniae* and methicillin-resistant *Staphylococcus aureus*. However, higher dosages were required for the treatment of SSTI, especially in obese patients.

Eleven out of 32 patients in our cohort were receiving CRRT whilst being treated with high-dose TGC. Interestingly, in a recent paper, *Broeker and cow* [20] described the PK/PD of standard-dose TGC in eleven patients on continuous veno-venous haemodialysis (CVVHD) or haemodiafiltration (CVVHDF). TGC dialysability, as expressed by saturation coefficients (0.79 and 0.9 for CVVHD and CVVHDF, respectively), was very high, but the contribution of CRRT TGC clearance was minimal (about 2 L/h), compared with the total body clearance (18.3 L/h). Peak drug concentrations were below 1 mcg/mL and trough levels about 0.2 mcg/mL. The authors, considering the AUC0-24/MIC referral value for cIAI (6.96), observed that such target was accomplished in 88% of the case if MIC was ≤ 0.5. This result is quite different from our findings where lower MIC values are required to get the optimal PK/PD target.

Indeed, our results are in line with current available data, underlying the plus-value of increased dosages whilst treating critically ill patients especially with severe cIAI and SSTI. In addition, there is a high need of PK/PD data on TGC administered at higher than approved dosages, in light of the wide spread of increased resistance to TGC amongst Gram-negative rods and *Acinetobacter* spp. The first investigation on PK/PD of HD TGC derives from Ramirez et al. who conducted a randomized phase 2 trial to evaluate the clinical efficacy of two high-dosage regimen of TGC (75 mg bid and 100 mg bid) versus imipenem–cilastatin (1 g every 8 h) for the treatment of nosocomial pneumonia [8]. In the clinically evaluable population, clinical cure with TGC 100 mg bid was numerically higher than with 75 bid and imipenem–cilastatin (1 g every 8 h) (85% vs. 69.6% vs. 75%). Mean peak TGC concentration was about 1 mcg/mL, declining to less than 0.5 mcg/mL after 8 h, observing a safety profile comparable to that one known for the approved those. The only other study investigating the PK/PD of HD TGC profile was conducted by Borsuk-De Moor et al. in 37 ICU patients with severe infections [21]. The time–concentration curve was similar to our data, displaying a peak concentration about 1 mcg/mL and 12 h level below 0.5 mcg/mL. Interestingly, the authors developed a model which showed that no individual covariates may influence target concentrations, advising to modify TGC daily dosage according to pathogens type, susceptibility pattern and PK targets.

Tissue concentrations of antibiotics at the target site contribute to therapeutic effects: using plasma concentrations may frequently overestimate the target site concentrations and therefore clinical efficacy. This is the first study to report steady-state ELF percentage penetration of TGC administered 100 mg q12 after 200 mg LD. Considering the AUC_0-24_/MIC target of 4.96, our data show satisfactory pulmonary concentrations with potential clinical success in 100%–94% to 75%–41% of the cases treating bacteria with MIC of 0.12–0.25 mcg/mL to 0.5–1 mcg/mL, respectively (Fig. 2). These data confirm the results observed in healthy subjects by Conte et al., where the *C*_max_/MIC90, AUC/MIC90 ratios, T > MIC90 and extended serum and intrapulmonary half-lives following the standard regimen are favourable for the treatment of TGC-susceptible pulmonary infections [22]. Penetration ratio may be even higher when in presence of infected lungs. *Crandon* et al. demonstrated in infected and non-infected mice lungs that the baseline penetration ratio of 8.1 is incremented to 23.3 in case of *Acinetobacter* pneumonia [23]. Conversely, the majority of lung penetration occurs in alveolar cells, than in ELF, as suggested by Welte et al. in three cases of MDR lung infections [24]. Finally in a recent study on 58 healthy subjects treated with standard TGC dose, the ratio of ELF and AUC to total plasma concentration of tigecycline was 1.71 and 20.8, respectively [25].

Our study has several limitations. First, we adopted a single high-dose of tigecycline and we do not know if even higher dosages may result in better PK/PD profiles. Second, we measured only pulmonary tissue concentration trough ELF collection and we can only postulate the real tissue/plasma ratio for cIAI and SSTI which, additionally, accounted only for less than 50% of the cases. Third, our analysis focused on total TGC concentration rather unbound AUC_0-24_, due to the lack of clinical reliable breakpoint of *f*AUC_0-24_/MIC_90_. Fourth, we did not provide real MIC values and we only simulated a wide range (0.12–2 mcg/mL) to compute AUC/MIC ratios and PTA percentages. Fifth, we did not sampled BAL from most hypoxemic/most severely patients, introducing a bias in the final results. Finally, the sample size may be likely responsible of an under-estimated interindividual variability in the observed PK/PD profile.

## Conclusions

Our study is the first investigation where not only plasmatic but also pulmonary tigecycline concentrations are investigated during the treatment of severe infections in critically ill patients with high-dose TGC. Observed plasmatic concentrations suggest the efficacy of this molecule for the treatment of susceptible pathogens, including pneumonia. Higher than 200 mg/day dosages and combination with other active molecules may be suggested whilst treating *Enterobacteriaceae* and *Acinetobacter* spp. with MIC values close to the clinical breakpoint, especially in case of SSTI and IAI.

## Supplementary information

**Additional file 1.** Sample collection.

## Data Availability

The datasets used and/or analysed during the current study are available from the corresponding author on reasonable request.

## Abbreviations

HD: High dosages
TGC: Tigecycline
PK/PD: Pharmacokinetic/pharmacodynamic
ICU: Intensive Care Unit
ELF: Epithelial lining fluid
AUC: Area under the curve
MIC: Minimum inhibitory concentration
IQR: Interquartile range
MRSA: *Staphylococcus aureus*
VRE: Vancomycin-resistant enterococci
ESBL: Extended-spectrum β-lactamase
XDR: Extensively drug-resistant
FDA: Food and Drug Administration
CRRT: Continuous renal replacement therapy
SAPS II: Simplified Acute Physiology Score
LD: Loading dose
DHHS-CTCAE: Department of Health and Human Services–Common Terminology Criteria for Adverse Events
VAP: Ventilator associated pneumonia
BAL: Bronchoalveolar lavage
IS: Internal standard
SPE: Solid-phase extraction
TQD: Triple quadrupole tandem
ESI: Electrospray ionization
MRM: Multi reaction monitoring
MS: Mass spectroscopy
*C*_max_: Maximum concentration
*C*_min_: Minimum concentration
Vd: Distribution volume
CL: Drug clearance
*t*1/2: Elimination half-life
IAI: Intra-abdominal infection
SSTI: Skin–soft-tissue infection
MALDI-TOF: Matrix-assisted laser desorption ionization-time-of-flight
EUCAST: European Committee on Antimicrobial Susceptibility Testing
SOFA: Sequential Organ Failure Assessment
ARF: Acute respiratory failure
AKI: Acute kidney injury
MV: Mechanical ventilation
cIAI: Complicated intra-abdominal infection
cSSTI: Complicated skin and soft tissue infection
CVVHD: Continuous veno-venous haemodialysis
CVVHDF: Continuous veno-venous hemodiafiltration
