# Supplementary material for: Pharmacokinetics of high-dose tigecycline in critically ill patients with severe infections
Source: Ann Intensive Care. 2020 Jul 13;10:94. doi: 10.1186/s13613-020-00715-2 (PMC7357259; doi:10.1186/s13613-020-00715-2)
Supplement: Supplementary file 1 — Additional file 1. Sample collection. [file 13613_2020_715_MOESM1_ESM.docx]

**5Additional file**

**Pharmacokinetics and intrapulmonary concentrations of high-dose tigecycline in critically ill patients with severe infections**

*Sample preparation*

Tigecycline liquid/liquid extraction from plasma samples

Liquid/liquid extraction was employed for purification and concentration of TGC from plasma samples. Briefly 25 μl of 0.1 mg/ml IS and then 50 μl of mobile phase A (H_2_O 0.1% HCOOH) were added to 250 μl of the plasma sample. After a rapid vortex mixing, 2.5 ml of Methyl-tert butyl ether was added to all samples, the mixture was vortexed for 5 min and then centrifuged at 3200 rpm for 10 min at 10 °C. The upper organic layer was separated and evaporated to dryness at 40 °C. The residue was then reconstituted in 100 μl of mobile phase B [ACN (+0.1 %HCOOH)] solution and transferred to an auto-sampler vial.

Tigecycline solid-phase extraction from BAL samples

Purification and concentration of TGC from BAL samples were carried out by solid-phase extraction (SPE). Briefly, 500 µL of BAL samples were mixed to 500 µL of 1%TFA in water, and 6 µL of concentrated TFA were subsequently added. After 5 min of vortex, the samples were centrifuged at 2500 rpm for 10 min; thereafter, the surnatants were collected and loaded onto SPE cartridges (OASIS HLB 1cc; Waters) arranged on an extraction manifold (Vac-Elut, Analytichem International). The SPE cartridges were sequentially pre-conditioned with 1 ml of MeOH given twice, and subsequently with 1 ml of water given twice. The loaded cartridges were washed with 1 ml of 1%TFA in water, and eluted with 1 ml of MEOH. The eluted solutions were evaporated to dryness at 40°C. The residues were reconstituted in 100 µl of {ACN (+0.5 %HCOOH): MeOH (+0.5% HCOOH) [70:30]} solution and then transferred to an auto-sampler vial

Chromatographic and Mass-Spectrometric Conditions

The chromatographic separation was performed on an AQUITY UPLC system (Waters Corp., Milford, MA, USA) with cooled auto-sampler and column oven for temperature control. An AQUITY UPLC BEH C18 column (1.7 um; 2.1 x 50 mm; Waters Corp., Milford, MA, USA) was employed and the column temperature was maintained at 40 °C. The elution was performed at a flow rate of 0.6 mL/min with a run time of 3 min. The UPLC was connected to a triple quadrupole tandem mass detector (TQD) (Waters Corp., Milford, MA, USA) with an electrospray ionization (ESI) source for the mass spectrometric detection. The ESI source was set in positive ion mode and the quantification was performed using multi reaction monitoring (MRM) mode for the most suitable mass transitions. The optimal mass spectroscopy (MS) parameters were showed in table 1. Data were processed using MassLynx software with a QuanLynx program version 4.1 (Waters Corp., Milford, MA, USA).
